# The prevalence and incidence of active syphilis in women in Morocco, 1995-2016: Model-based estimation and implications for STI surveillance

**DOI:** 10.1371/journal.pone.0181498

**Published:** 2017-08-24

**Authors:** Aziza Bennani, Amina El-Kettani, Amina Hançali, Houssine El-Rhilani, Kamal Alami, Mohamed Youbi, Jane Rowley, Laith Abu-Raddad, Alex Smolak, Melanie Taylor, Guy Mahiané, John Stover, Eline L. Korenromp

**Affiliations:** 1 Ministry of Health, Directorate of Epidemiology and Disease Control, Rabat, Morocco; 2 STIs Laboratory, Department of Bacteriology, National Institute of Hygiene, Rabat, Morocco; 3 UNAIDS Morocco country office, Rabat, Morocco; 4 London, United Kingdom; 5 Weill Cornell Medical College - Qatar, Cornell University, Doha, Qatar; 6 World Health Organization, Dept. of Reproductive Health and Research, Geneva, Switzerland; 7 Centers for Disease Control and Prevention, Division of STD Prevention, Atlanta, Georgia, United States of America; 8 Avenir Health, Glastonbury, Connecticut, United States of America; Columbia University, UNITED STATES

## Abstract

**Background:**

Evolving health priorities and resource constraints mean that countries require data on trends in sexually transmitted infections (STI) burden, to inform program planning and resource allocation. We applied the Spectrum STI estimation tool to estimate the prevalence and incidence of active syphilis in adult women in Morocco over 1995 to 2016. The results from the analysis are being used to inform Morocco’s national HIV/STI strategy, target setting and program evaluation.

**Methods:**

Syphilis prevalence levels and trends were fitted through logistic regression to data from surveys in antenatal clinics, women attending family planning clinics and other general adult populations, as available post-1995. Prevalence data were adjusted for diagnostic test performance, and for the contribution of higher-risk populations not sampled in surveys. Incidence was inferred from prevalence by adjusting for the average duration of infection with active syphilis.

**Results:**

In 2016, active syphilis prevalence was estimated to be 0.56% in women 15 to 49 years of age (95% confidence interval, CI: 0.3%-1.0%), and around 21,675 (10,612–37,198) new syphilis infections have occurred. The analysis shows a steady decline in prevalence from 1995, when the prevalence was estimated to be 1.8% (1.0–3.5%). The decline was consistent with decreasing prevalences observed in TB patients, fishermen and prisoners followed over 2000–2012 through sentinel surveillance, and with a decline since 2003 in national HIV incidence estimated earlier through independent modelling.

**Conclusions:**

Periodic population-based surveys allowed Morocco to estimate syphilis prevalence and incidence trends. This first-ever undertaking engaged and focused national stakeholders, and confirmed the still considerable syphilis burden. The latest survey was done in 2012 and so the trends are relatively uncertain after 2012. From 2017 Morocco plans to implement a system to record data from routine antenatal programmatic screening, which should help update and re-calibrate next trend estimations.

## Introduction

Sexually transmitted infections (STIs) constitute a public health burden in Morocco as elsewhere. Around 400,000 new cases are registered through public health clinics every year, but the true burden is believed to be higher as cases that are not symptomatic and not treated, or which are managed by private health providers or self-treated are not reported. Of reported STI cases, 3% are genital ulcers (and the remainder reported as: vaginal discharge, urethral discharge (UD), condylomata, hepatitis and other STI); 75% are in women; and 65% are in young adults aged 20–40 years [1].

Morocco adopted the syndromic STI treatment approach in 1998 and extended it at national level in 2000, with a nation-wide training program of health providers [2]. Both case management algorithms and therapeutic protocols are regularly revised in response to periodic etiological studies and updated global guidelines. Within the framework of the National AIDS Program, a specific area of intervention has been strengthening the management of STIs and integrating prevention of HIV/AIDS within Reproductive Health services, both in public health and in non-governmental organization (NGO) facilities [1].

Morocco’s STI surveillance system includes: 1) STI cases notification: integrated into the routine activities of public health facilities and NGO-led centres; 2) Sentinel surveillance of syphilis in various population groups, from 1993 to 2012; (3) Integrated Bio-Behavioural Surveys (IBBS) among most at risk populations; (4) periodic prevalence, case etiology and drug resistance studies; and 5) Behavioural and bio-behavioural studies.

Sentinel surveillance was halted after 2012; it will be replaced in the near future by a system for continuous collection of routine data from syphilis screening among pregnant women visiting antenatal care clinics (ANC). IBBS and prevalence/etiology and resistance studies have been supported by international donor financing, notably the Global Fund to Fight AIDS, Tuberculosis and Malaria; the World Health Organization (WHO), and the United Nations Population Fund (UNFPA). However, reductions in external donor budgets may seriously compromise the STI surveillance system in Morocco, one of the most advanced national surveillance systems in the Middle East and Africa region [3, 4].

In 2016, the World Health Assembly and its member states adopted the WHO’s Global Health Sector Strategy on STIs, 2016–2021 [5]. The strategy sets an impact target, from 2018 to 2030, a 90% incidence reduction for syphilis and gonorrhea [5]. Syphilis is an area of special focus, considering the morbidity and mortality burden associated with untreated maternal and congenital syphilis. Despite the fact that syphilis in pregnancy is relatively simple and inexpensive to diagnose and treat with penicillin, the WHO estimated that 1.36 million pregnant women globally had probable active syphilis in 2008; leading to 520,905 adverse pregnancy outcomes [6]. These numbers formed the baseline for the WHO’s initiative to eliminate mother-to-child transmission of syphilis, and numerous countries from all regions, including Morocco, have committed to the elimination of congenital syphilis [7].

The Spectrum STI estimation model was developed in 2016 as a tool that national STI programs and the WHO could use to estimate temporal trends in prevalence and incidence of STIs (syphilis, gonorrhea and chlamydia) and to monitor progress in STI control, including the elimination of congenital syphilis. Spectrum-supported STI trend estimation by national program managers and surveillance staff supports usage and quality checking of surveillance data, and informs data collection priorities going forward, thereby strengthening local surveillance processes, systems and capacity [8].

This article introduces the Spectrum methodology for estimating syphilis incidence based on syphilis prevalence, and applies the estimation for Morocco as a pilot country.

Secondary aims of this analysis were:

- To assemble, document and synthesize available syphilis prevalence data from sentinel surveillance and integrated bio-behavioural surveys from Morocco, and to triangulate prevalence trend data in key population groups monitored, with syphilis trends estimated for the national population;
- To compare Spectrum-estimated syphilis trends with estimated trends in HIV incidence generated earlier and independently by Morocco’s HIV/STI program using the (independent) Spectrum-based HIV transmission model called Goals [9, 10], and to compare and interpret estimated time trends of HIV and syphilis incidence in view of their shared underlying risk factors;
- Based on the syphilis trend estimates, propose strategies to strengthen STI surveillance in Morocco.

## Methods

### Overview

The Spectrum-STI epidemiological projection tool was used to estimate and project the prevalence of active syphilis in adult women (age 15–49 years) in Morocco between 1995 and 2021 based on statistical fitting of data from surveys conducted in pregnant women attending ANC or family planning services [8]. The Spectrum-STI tool is embedded in a broader suite of demographic and health burden and impact modelling program projection tools used by over 120 countries to estimate the burden of HIV/AIDS and the associated need for HIV/AIDS treatment. The software is available free of charge at: http://avenirhealth.org/software-spectrum.php.

The data and assumptions used to generate the Morocco estimates were reviewed, discussed and agreed during two technical workshops, held in May and September 2016. Participants at these meetings included representatives of Morocco’s Ministry of Health and its provincial offices, HIV/AIDS and Maternal and Child Health programs; Morocco’s central reference laboratory, the World Health Organization and UNAIDS, and other partners supporting or implementing the national HIV/STI response. All data collated had been collected and documented earlier, independently from the current study, by Morocco’s HIV/STI program and HIV/STI surveillance unit within the Ministry of Health.

### Prevalence estimation

Active syphilis infection was defined as a patient positive concurrently on both the Rapid Plasma Reagin (RPR) test and the Treponema pallidum haemagglutination assay (TPHA) test [11]. The details of the model used to generate the syphilis prevalence trends are described in detail in [8]. In brief, syphilis prevalence data from surveys and routine programmatic screening in pregnant women presenting for ANC, women attending family planning (FP) clinics, and any general adult population surveys that met the criteria for representativeness and quality used in the WHO 2012 regional and global estimations [11] were collated. Prevalence estimates were adjusted for the diagnostic test used: against the gold standard of dual RPR and TPHA positivity, which was taken as observed, prevalences based on RPR-positivity regardless of TPHA status were adjusted downward to 0.7 of the observed prevalence for ANC and FP populations; or to 0.6-fold for other general populations; prevalences based on TPHA-positivity regardless of RPR status were adjusted to 0.8-fold the observed value regardless of the population, prevalences based on rapid TPHA-based test in an ANC population to 0.7-fold; and prevalences based on an unknown test to 0.75-fold [8, 12].

Test-adjusted prevalence data were then adjusted upward by 10% to account for under-sampling of high-risk populations in general population surveys, also based on WHO’s 2005, 2008 and 2012 regional and global estimations methodology [11].

Each data point was then assigned a weight, reflecting its national representativeness. For Morocco’s ANC surveys, this weight was calculated by dividing the number of ANC sites sampled in a particular year by 30, the maximum number of ANC sites (in 2009). For example, the 1996 survey, with 9 sites, was given a 30% (= 9/30) weight. Time trends were fitted by logistic regression through all data points combined, assuming no systematic prevalence differences between ANC women, FP women, other general adult women, or general adult men, similar to the approach of the WHO’s regional and global estimations [11, 13, 14]. 95% confidence intervals (CI) were generated by bootstrapping, and the median result across 10,000 bootstrapping iterations taken as the best estimate [8].

### Estimation of incidence from prevalence

Incidence was derived from the prevalence trend estimates. We assumed that the incidence hazard or density (among uninfected people) was constant in each of the consecutive intervals of length 1 year, for *t* starting from 1995. If the incidence hazard or density, *i*, and the duration of the STI disease episode, *D*, are constant in an interval (*t*_0_, *t*_0_ + 1), then for all *t* in that interval, the prevalence satisfies the equation:

$$p\left(t\right)=\frac{i}{i+r}+\left(p\left(t_{0}\right)-\frac{i}{i+r}\right)\text{exp}\left(-\left(i+r\right)\left(t-t_{0}\right)\right)$$ (1)

where *r* = 1/*D*.

We solved this equation for *i* piecewise every year, after setting *t* = *t*_0_ + 1, for *t*_0_ = 1995… to obtain its time trend. From this incidence hazard, the corresponding incidence rate ‘IR’ *per capita* in the overall national population was calculated as:*IR* = *i*(1 − *p*), where *p* is the prevalence (see S1 Text).

In the default estimation, the average duration of infection with syphilis was assumed to be constant over time and set at 2.42 years. This estimate was based on the values used by the WHO in their 2012 regional and global STI estimations for the Eastern Mediterranean region [11] (S1 Table [11]).

In an alternative scenario, the duration of infection was assumed to have progressively decreased over time, from 3.1 years in 1995 to 2.42 years in 2015. This change was made to reflect improvements in access to treatment. In 1995, we assumed 30% of individuals with symptoms were treated, and by 2015 this had increased to 60%, the value also assumed in the WHO 2012 estimations for the Eastern Mediterranean region. The assumed doubling of treatment coverage was in line with our recent Spectrum-STI-based estimation of Morocco’s gonorrhea and chlamydia trends, where we assumed that the treatment coverage of symptomatic gonorrhea and chlamydia in men doubled from 1995 to 2015 [15]. The latter is was based on treatment coverage reported through Knowledge, Attitudes and Practices surveys in youth [16, 17] and among men with urethral discharge [18, 19]. Reasoning that the improved treatment coverage for UD reported in these surveys reflected a general improvement in STI clinical services, we assumed here that treatment coverage also doubled for syphilis. Applying the durations of untreated syphilis and of treated syphilis (S1 Table) to the proportions that are treated and untreated (S1 Table), yielded a weighted average duration of infection that decreased from 3.1 years at 1995 to 2.42 years at 2015.

We assumed no disease mortality for adult syphilis.

The 95% CIs on incidence bounds reflected the uncertainty (estimated by bootstrapping) in prevalence, and an additional uncertainty on the duration of infection, set at ±50%.

### Triangulation of national syphilis estimates with syphilis trends in key populations

For comparison with the Spectrum-estimated trends based on ANC, FP and general population surveys, we compiled syphilis prevalence data from sentinel surveillance and integrated bio-behavioural surveys collected since 1995 in Morocco, as available at the Ministry of Health.

### Triangulation with national HIV estimates and modelled sexual risk behavioural trends

To support interpretation of estimated syphilis trends, we assessed time trends in HIV incidence and in risk behaviours underlying HIV incidence, estimated using the HIV transmission model Goals, another module of the Spectrum suite [10]. The representation of Morocco’s HIV epidemic and its drivers in the Goals model was that used in a recent multi-country modelling of the UNAIDS global Fast Track targets [9]. For Morocco, the model representation had been informed by analysis of data from Morocco’s sentinel surveillance and IBBS data, two Modes-of-Transmission studies [4, 20, 21] by the Ministry of Health with the UNAIDS country office, and Morocco’s 2015 round of annual HIV burden estimation that had used the Spectrum module AIDS Incidence Model [22].

## Results

### Estimated syphilis prevalence

**Fig 1:**
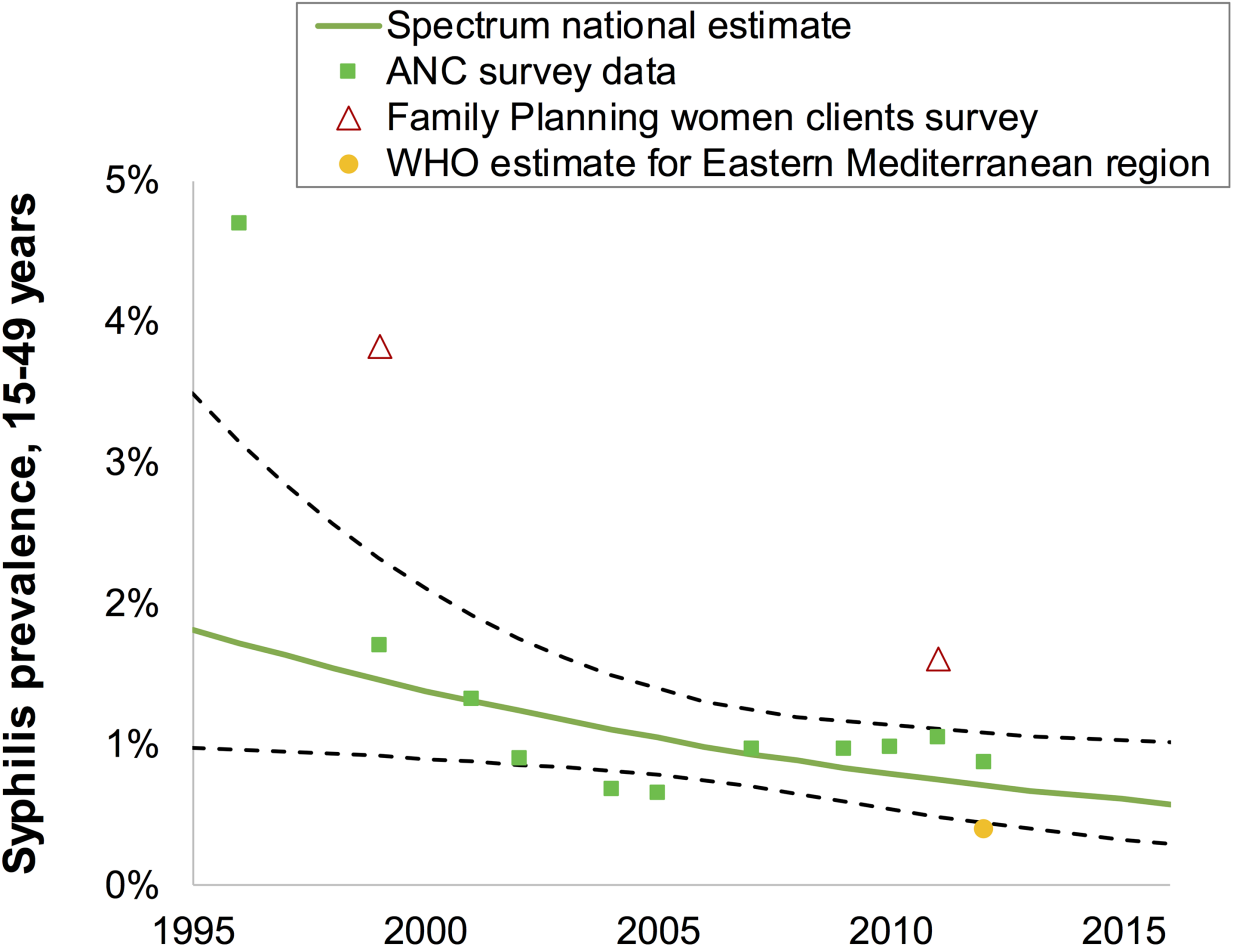
Spectrum-estimated national syphilis prevalence, women 15–49 years, Morocco. Data are shown after adjustments for diagnostic test performance, and missing high-risk populations, as described in the Methods. Dashed green lines are 95% Confidence Intervals around the estimate.

Table 1 and Fig 1 record all syphilis studies that met the study entry criteria. These included 10 ANC surveys conducted between 1996 and 2012, and two small-scale surveys among women attending FP clinics in 1999 and 2011–2012. The data from both ANC and FP populations suggest a declining syphilis prevalence over time (Fig 1). Based on these data, the estimated syphilis prevalence declined from 1.8% (1.0–3.5%) in 1995 to 0.57% (0.3–1.0%) in 2016.

**Table 1:** Prevalence data used, and adjustments for diagnostic test performance, and missing high-risk populations, in the Spectrum-STI estimation of syphilis prevalence and trend for adult (15–49 years) women in Morocco.

Year	Population & sites	N	TPHA+	TPHA%	RPR+	RPR%	TPHA+ RPR+	TPHA+ RPR+ %	Diagnostic test, data point used	Test-adjusted	High-risk adjusted	Weight^$^
1996	ANC sentinel, 9 sites	2,459			175	**7.1%**			RPR	4.3%	4.7%	30%
1999	ANC, Rabat/ & Salé (2 sites) [23]	323					5	**1.55%**	RPR+TPHA	1.55%	1.70%	6.7%
1999	FP, Rabat & Salé (2 sites) [23]	518					18	**3.50%**	RPR+TPHA	3.50%	3.80%	6.7%
1999	ANC sentinel, 9 sites*	10,135	113	1.11%	154	**1.52%**			TPHA*	0.89%	0.98%	30%
2001	ANC sentinel, 9 sites	3,154			63	**2.00%**			RPR	1.20%	1.32%	10%
2002	ANC sentinel, 19 sites	16,666			225	**1.35%**			RPR	0.81%	0.89%	63%
2004	ANC sentinel, 23 sites	18,302			187	**1.02%**			RPR	0.61%	0.67%	77%
2005	ANC sentinel, 25 sites*	17,711	129	**0.73%**	148	1.00%			TPHA*	0.58%	0.64%	83%
2007	ANC sentinel, 27 sites*	16,422	179	**1.09%**	161	1.13%			TPHA*	0.87%	0.96%	90%
2009	ANC sentinel, 30 sites*	15,290	166	**1.09%**	135	1.01%			TPHA*	0.87%	0.96%	100%
2010	ANC sentinel, 6 high-HIV sites	3,147	35	**1.11%**	26	1.11%			TPHA*	0.89%	0.98%	20%
2011–2012	ANC; Agadir, Fes, Rabat, Salé &Temara [24]	252			4	**1.59%**			RPR	0.95%	1.05%	17%
2011–2012	FP, Agadir, Fes, Rabat, Salé & Temara [24]	537			13	**2.42%**			RPR	1.45%	1.60%	17%
2012	ANC sentinel, 18 sites	7,981	78	**0.98%**	53	0.44%			TPHA*	0.78%	0.86%	60%

Abbreviations: ANC = antenatal clinic attendants; FP = family planning clinic (female) clients; RPR = rapid plasma reagin; TPHA = *Treponema pallidum* hemagglutination assay; N = sample size tested; Test-adjusted = prevalence after adjusting for diagnostic test sensitivity & specificity; PHC = Primary Health Care clients; High-risk adjusted = prevalence after (+10%) adjustment for missing high-risk populations.

^$^ Weight = statistical weight used in the Spectrum trend estimation; calculated by dividing the number of ANC sites sampled by 30, the maximum number of ANC sites sampled (in the 2009 survey). For example, the 1996 survey with 9 sites was given a 30% (= 9/30) weight.

* TPHA and RPR were measured in these ANC surveillance rounds, but combined (TPHA+/RPHR+) prevalence was not recorded. For Spectrum-STI fitting, the TPHA prevalence in ANC women was judged more relevant than the RPR prevalence, because of higher sensitivity. Prevalences based on TPHA-positivity alone (as well as those based on RPR-positivity alone) were adjusted for likely over-diagnosis of active syphilis, compared to the gold standard of dual TPHA+/RPR+ positivity, as described in the Methods section.

### Estimated syphilis incidence

**Fig 2:**
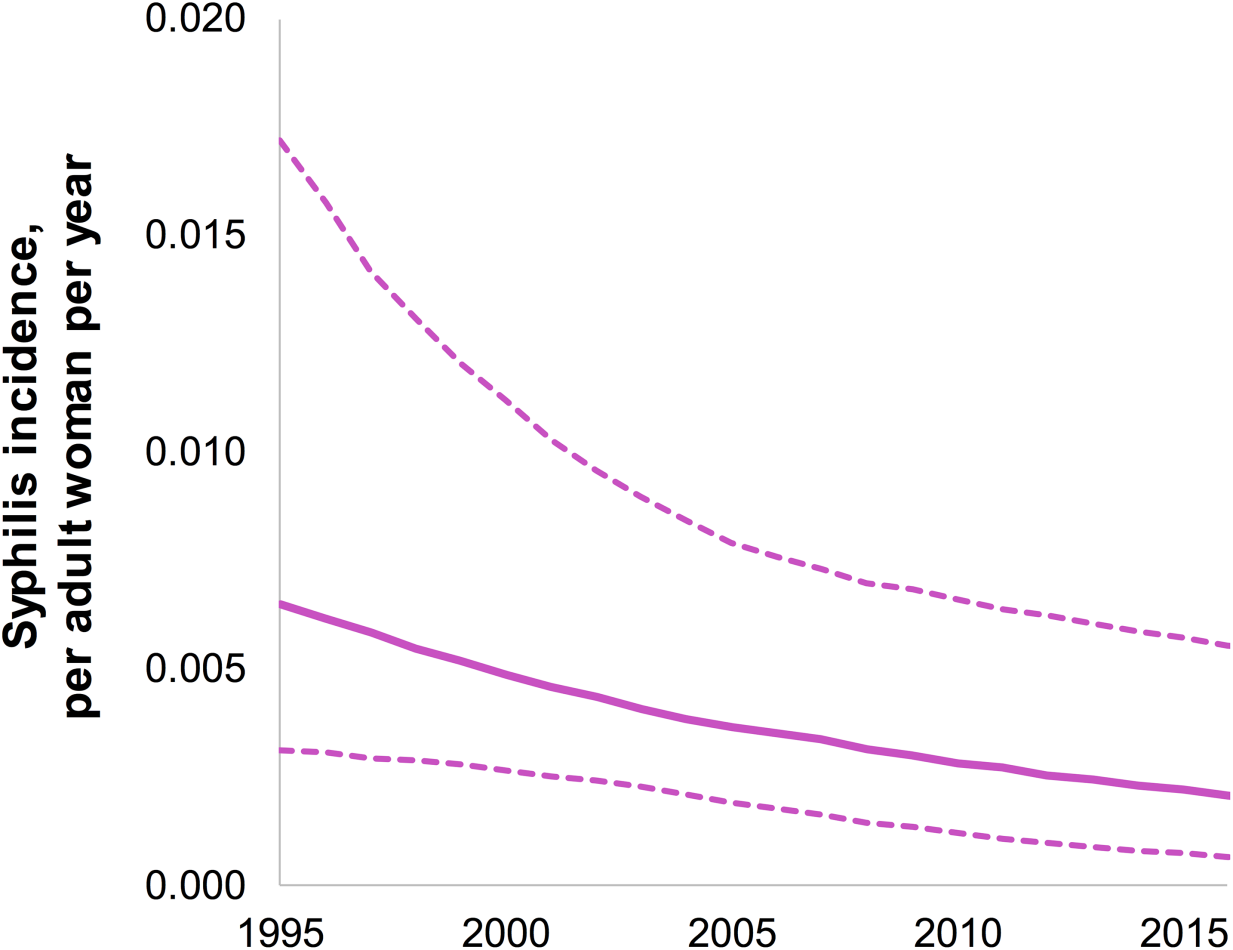
Spectrum-estimated syphilis incidence rates in women 15–49 years, Morocco. Dashed lines are 95% Confidence Intervals. The estimation shown assumed a constant duration of syphilis episodes over time, reflecting a time-constant coverage of treatment (which is the default assumption in the Spectrum tool).

Fig 2 shows syphilis incidence rates, based on the estimated prevalence levels and trends. Based on these prevalence estimates from Spectrum-STI, the estimated incidence rate was 200 per 100,000 adult women, and there were an estimated 21,675 (95% CI 10,612–37,198) new syphilis cases in women in 2016 (Table 2).

**Table 2:** Spectrum-estimated prevalence and incidence rate (per 100,000 person-years) of active syphilis in 2016 in women 15–49 years, Morocco.

Metric	2016 best estimate	95% confidence interval
Prevalence	0.57%	0.28%–0.98%
Incidence hazard per 100,000 uninfected adult women	200	63–576
Incidence rate per 100,000 total adult women	199	63–570
New incident cases, women 15–49 years	21,675	10,612–37,198

Under the alternative scenario that assuming a doubling of syphilis treatment coverage over 1995–2015, with the resulting longer average episode duration in pre-2016 years when treatment coverage had been lower, the estimated incidence rate in 1995 was 450 per 100,000 adult women (compared to 660 per 100,0000 adult women in the default estimation, Fig 2).

In the default estimation (with time-constant treatment coverage and duration), estimated incidence declined by 67%, from 660 per 100,000 in 1995 to 200 per 100,000 in 2016 (Fig 2). In the alternative estimation (with improving treatment coverage and shortening duration), the estimated incidence declined by 44%, from 580 to 200 per 100,000, over this period.

### Prevalence trends in selected population groups in sentinel surveillance

**Fig 3:**
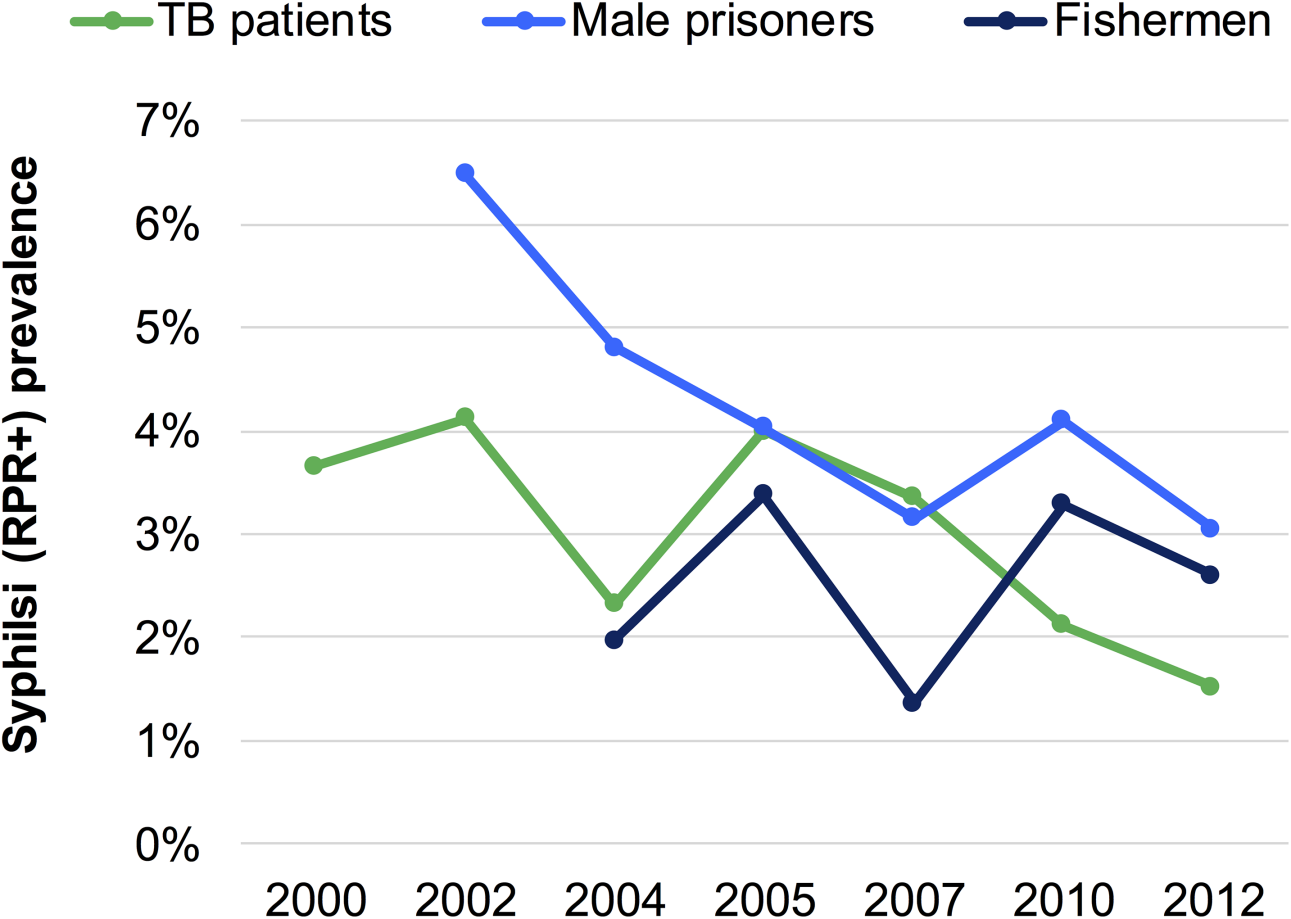
Prevalence of RPR positivity, sentinel surveillance in Morocco. Data shown are for RPR positivity regardless of TPHA status. For some of these years and population groups, TPHA was also measured, but the dual combined RPR/TPHA status was not recorded. Since there were more data points for RPR than for TPHA, the current figure shows the RPR results. For sample sizes, numbers testing positive and corresponding TPHA positivity results, see S2 Table.

Fig 3 shows time trends in prevalence of RPR positivity (without TPHA confirmation) from sentinel surveillance in those populations for which there were at least 5 points in time. Syphilis prevalence was stable or fluctuating in tuberculosis (TB) patients and fishermen, while it fell over 2002–2012 in male prisoners. No data were available after 2012, which was the last year that sentinel surveillance was conducted.

Additional surveillance data of RPR positivity over 2000–2012 are available for STI patients, female sex workers (FSW), men who have sex with men (MSM), female prisoners, injecting drug users (IDU), as well as for various groups of adult workers including truckers and seasonal workers (S2 Table). Fig 3 does not include trends for these population groups, because they are less representative of the general adult population, because they had fewer than 5 data points within the 1995–2015 time period, and/or because the sites and samples taken for these groups were not considered comparable enough over subsequent surveillance rounds to validly inform the time trend.

### Time trends in HIV incidence, and underlying sexual risk behaviours in Morocco

**Fig 4:**
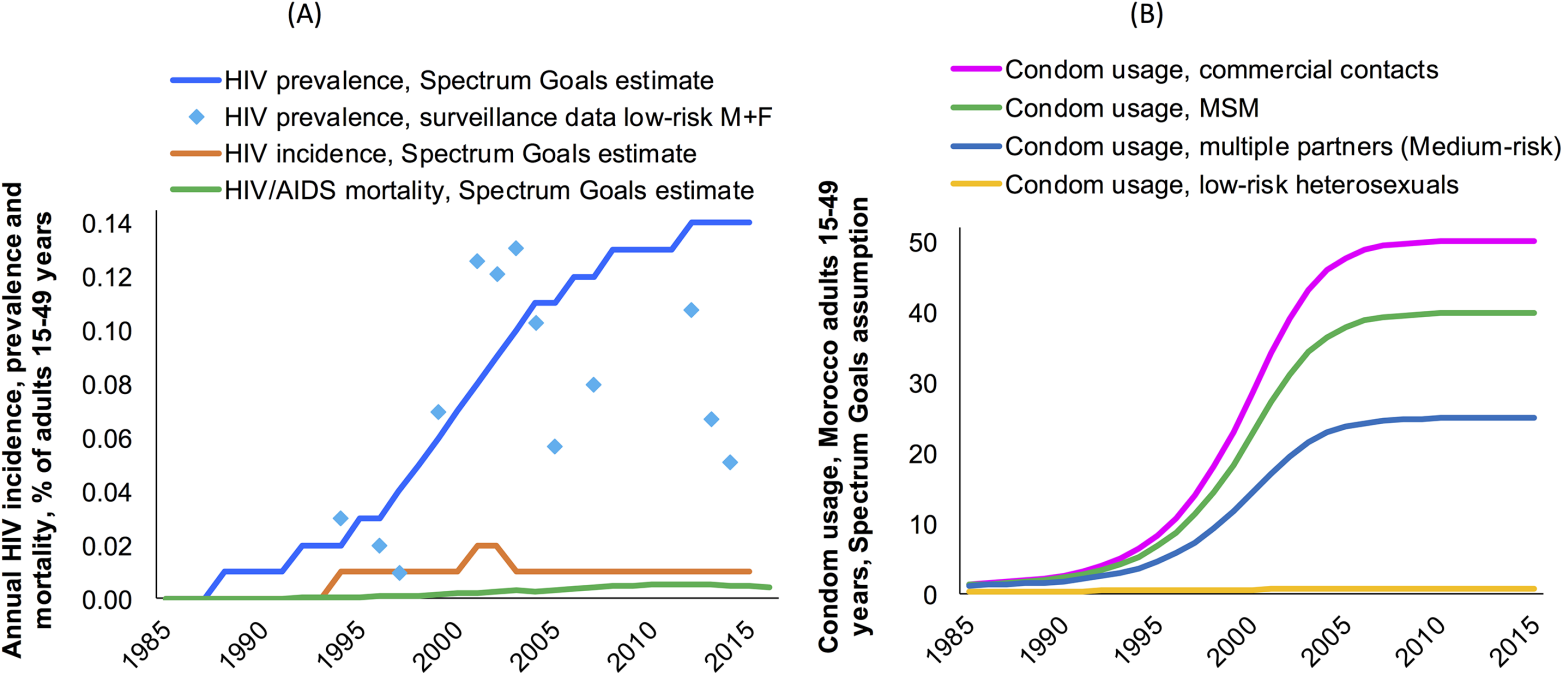
Time trends in (a) HIV incidence, prevalence and mortality and (b) condom usage, in Morocco. Epidemiological estimates and condom usage assumptions from the Spectrum Goals model. HIV incidence rates shown are the hazard/density, within the uninfected population. Abbreviations: MSM = Men who have sex with men; F = women; M = men.

Estimations by the Spectrum Goals model show HIV incidence peaking around 2002 and subsequently stabilising at a lower level (Fig 4a), reflecting changes in sexual behaviour including increased condom use over 1995–2003 (Fig 4b). These results are consistent with the syphilis projections. Morocco’s Spectrum-predicted syphilis prevalence decline over 1995–2015 is consistent with these independent earlier estimates of a reduction in sexual risk behaviour, reflecting empirical data indicating increasing condom use particularly among populations at high risk such as female sex workers [4, 20–22], and increasing volumes of condom distribution by Non-Governmental Organizations since 2004, which explained not only a decline in HIV incidence since 2003, but which also likely contributed to declining syphilis incidence.

## Discussion

The Spectrum STI model provides a tool to look at population-based surveys in a systematic manner to estimate STI rates and their time trends. Morocco, as a pilot country, used the model to estimate recent syphilis prevalence and incidence trends in adult women based on survey data collected starting from 1995. This first-ever undertaking engaged and focused national stakeholders, and confirmed the still considerable syphilis burden.

The estimation results inform and support Morocco’s National HIV/AIDS and STI Plan and National Health Strategy [1], through which the Ministry of Health continues to include control of STIs, and particularly syphilis, among its public health priorities. Additionally, the Ministry of Health continues its commitment to the elimination of congenital syphilis, in line with the global target [7].

On the positive side, Spectrum estimated a decline in Morocco’s incidence and prevalence of active syphilis over 1995–2016. While the estimation was based on data primarily from ANC women, the estimated decline in adult women prevalence is consistent with the declines observed in prisoners and TB patients. A similar analysis focused on gonorrhea and chlamydia infections in adults in Morocco using the Spectrum approach also reported a decline in prevalence and incidence between 1995 and 2015 [15, 25].

The estimated decline in the prevalence and incidence of adult syphilis is further supported by independent earlier estimates [9] of declines in Morocco’s HIV incidence from a peak in 2002, and improvements in both condom usage and treatment seeking behaviour for STIs [4, 20–22]. In fact, the decline in syphilis (and other STIs) since 1996, may have contributed to the more recent HIV incidence decline, since STIs including syphilis are biological cofactors for HIV transmission [26]. The Spectrum syphilis estimation suggests that prevalence and incidence of syphilis continue to decline in Moroccan women. This is different from HIV, where incidence appears to have stabilized around 2005 [22], although given the very low levels of HIV incidence in Morocco this is very sensitive to small changes in risk behaviours among key groups, and the estimation method by which HIV incidence in past years is a back-calculation. For syphilis, the ongoing decline appears plausible considering Morocco’s adoption of syndromic management since 2001 whereby all genital ulcers provisionally receive syphilis treatment, and the more recent (still incomplete) roll-out of routine ANC-based syphilis screening and treatment

Morocco’s estimated syphilis prevalence of 0.71% at 2012 is higher than that estimated by the WHO for adult men and women in the Eastern Mediterranean region in 2012, of 0.39% (95% CI: 0.18–1.00%) [11], and also higher than the WHO estimate for ANC women in this region over 2009–2012 of 0.37% [27]. While the estimates are within the confidence limits of each other, the difference may relate to Morocco’s relatively slow roll-out of routine ANC syphilis screening, due to relatively low ANC attendance (67% with one or more visits to a skilled provider, at 2003–2004 [28]), insufficient availability of rapid diagnostic tests in ANC settings and other point-of-care clinics, and the user fees maintained for rapid tests at referral laboratories.

Both the current national, and the 2005, 2008 and 2012 WHO regional syphilis estimations [11] assumed a crude, fixed 10% upward prevalence adjustment for high-risk populations typically missing from population prevalence surveys. The 10% adjustment value was based on meta-analysis of comparative syphilis prevalence and population sizes of FSW relative to low-risk women in multiple countries. In Morocco comparative syphilis prevalences in FSW (13.4%) relative to low-risk women (2.4%) measured in a 2007 survey [29], combined with population sizes of 75,000 FSW [4, 21, 30] and 8 million low-risk women 15–49 years suggest a 4% contribution of sex workers, which supports the default 10% upward adjustment, given that other additional higher-risk groups besides FSW would further contribute to a prevalence somewhat higher than that sampled through ANC and FP surveys.

Estimated incidence rates are less certain than estimated prevalence, since there is an additional uncertainty arising from our imprecise knowledge of the duration of infection and, compounding that, uncertainty on how the average duration of infection may have changed over the estimation time horizon in response to changes in the coverage of treatment. Our results illustrate how estimated incidence in 1995, and the extent of incidence decline over 1995–2015 varies in a range depending on assumptions of the historic treatment coverage. As yet, data are lacking on Morocco’s nation-wide coverage of ANC-based and other syphilis screening and treatment programs, precluding a precise quantification of the relative contribution of the two probable contributing causes of the observed prevalence decline: decreasing duration thanks to improving treatment coverage, and decreasing incidence. Once data from ANC-based syphilis programs become available, they can be used to refine the syphilis incidence rate estimation. Meanwhile, it may be reasonable to assume that syphilis treatment coverage improved in Morocco since 1995, similar to improvements in treatment coverage for UD [15], and considering that syphilis cure rates likely increase with increasing use of antibiotics for other (non-STI) conditions (e.g. doxycycline and macrolides such as azithromycin, commonly prescribed for skin and respiratory infections) [31], as these drugs are generally also effective against syphilis.

Although syphilis incidence at 1995 (450 to 660 per 100,000 adult women), and the decline in this incidence over 1995–2015 (44% to 67%) thus remains relatively uncertain, the presented range nevertheless provides confidence that syphilis incidence rate must considerably have declined in Morocco in the period 1995–2015.

In view of the global impact target set in the WHO’s Global Health Sector Strategy on STIs 2016–2021, of reducing syphilis incidence by 90% from 2018 to 2030 [5], and since national programs need incidence estimates to evaluate their coverage and completeness of STI case reporting, we believe that the case incidence estimation merits further consideration and refinement. Besides representative prevalence data, this requires national estimates of syphilis treatment coverage (in women and men), and better estimates of the duration of treated and untreated infections.

The current study did not estimate syphilis rates in men, from whom there are typically not enough prevalence data. The Spectrum-STI modelling tool is able to generate predictions for male prevalence and incidence rates and numbers for adult men, derived by applying the same prevalence and duration of infection estimates in men as in women. In Morocco, this approach would imply very similar numbers of prevalent and incident infections in men as in women. Equal prevalence in men and women was an assumption in the WHO 2012 regional and global STI estimations [11], and appears to be supported for Morocco by an average male-to-female prevalence ratio of about one found in tuberculosis patients in sentinel surveillance data over 2005–2012 (of 1.06 for TPHA and 1.00 for RPR) [32]. While male prevalence was measured in Morocco in sentinel surveillance in prisoners, MSM, truck drivers and fishermen, samples were small, sites not nationally representative (S2 Table), and extrapolation from these population groups to the overall male population is not straightforward. The ideal data source to validate or refine assumed male syphilis prevalence and male-to-female prevalence ratios (as well as age patterns in prevalence, ignored in the current estimations) is population-based surveys including adult men and women alongside. Such data are available from some sub-Saharan African countries, from selected Demographic and Health Surveys, and most recently through national HIV surveys conducted over 2016–2018, with support of the United States of America’s Presidential Emergency Plan for AIDS Relief program [33]–but not for countries in the Eastern Mediterranean region.

Despite limitations, the estimates presented here are timely to inform Morocco’s 2017–2021 HIV/STI strategy, being developed in 2016. Since Morocco’s ANC surveillance ended in 2012, and Morocco is rolling out routine programmatic syphilis screening, it is imperative that these measurements be captured for surveillance purposes. An information system for this purpose has been launched in other countries, and is expected to be established in Morocco during 2017. Annual reporting of national-level results of routine programmatic syphilis screening in ANC services is recommended by the WHO, and ANC syphilis screening coverage and prevalence results included as core indicators in the Global AIDS Progress Response Reporting system to monitor and inform congenital syphilis prevention efforts [34–36].

In conclusion, the estimated adult female syphilis declines show that Morocco is progressing in its response against STIs. However, roll-out of syphilis screening and treatment, and recording and evaluation of prevalence data from routine ANC-based syphilis screening are needed to achieve and demonstrate congenital syphilis elimination.

## Supporting information

S1 Text Derivation of the incidence hazard and rate, from prevalence. (DOCX)

S1 Table Duration of adult syphilis infection, in years. (DOCX)

S2 Table Syphilis prevalence (RPR, without TPHA confirmation) in sentinel surveillance in non-ANC groups, Morocco. (DOCX)

## Abbreviations

ANC: antenatal clinic attendants
CI: confidence interval
F: women
FP: Family Planning clinic (female) clients
FSW: female sex worker(s)
GUD: genital ulcer disease
MSM: men who have sex with men
N: sample size tested
PHC: Primary Health Care
RPR: Rapid Plasma Reagin test
STI: sexually transmitted infection
TPHA: *Treponema pallidum* hemagglutination assay

## References

[1] Royaume du Maroc Programme National de lutte contre les IST/SIDA Direction de l'épidémiologie et des luttes contre les maladies du Ministère de la Santé. Le plan stratégique national de lutte contre le Sida (2012–2016). Rabat: 2012 4 April. Report No.

[2] Programme national de lutte contre les IST/SIDA, Ministère de la santé du Maroc Direction de l'Epidemiologie et de Lutte contre les Maladies. La prise en charge syndromique des infections sexuellement transmissibles. Guide du prestataire. Rabat: 1999.

[3] Kouyoumjian SP, Mumtaz GR, Hilmi N, Zidouh A, El Rhilani H, Alami K, et al The epidemiology of HIV infection in Morocco: systematic review and data synthesis. Int J STD AIDS. 2013;24(7):507–16. Epub 2013/08/24. doi: 10.1177/0956462413477971 . 23970764 10.1177/0956462413477971 PMC3764773

[4] Mumtaz GR, Kouyoumjian SP, Hilmi N, Zidouh A, El Rhilani H, Alami K, et al The distribution of new HIV infections by mode of exposure in Morocco. Sex Transm Infect. 2013;89 Suppl 3:iii49–56. Epub 2013/02/16. doi: 10.1136/sextrans-2012-050844 . 23413401 10.1136/sextrans-2012-050844 PMC3841748

[5] World Health Organization. Global health sector strategy on sexually transmitted infections 2016–2021. Towards ending STIs. Report. Geneva: 2016 June. Report No.: WHO/RHR/16.09.

[6] Newman L, Kamb M, Hawkes S, Gomez G, Say L, Seuc A, et al Global estimates of syphilis in pregnancy and associated adverse outcomes: analysis of multinational antenatal surveillance data. PLoS Med. 2013;10(2):e1001396 Epub 2013/03/08. doi: 10.1371/journal.pmed.1001396 . 23468598 10.1371/journal.pmed.1001396 PMC3582608

[7] World Health Organization. The global elimination of congenital syphilis: rationale and strategy for action. Geneva: 2007.

[8] Korenromp EL, Mahiané G, Rowley J, Nagelkerke N, Abu-Raddad L, Ndowa F, et al Estimating prevalence trends in adult gonorrhoea and syphilis prevalence in low- and middle-income countries with the Spectrum-STI model: results for Zimbabwe and Morocco from 1995 to 2016. Sex Transm Infect. 2017;(sextrans-2016-052953). doi: 10.1136/sextrans-2016-052953 28325771 10.1136/sextrans-2016-052953 PMC5739862

[9] Stover J, Bollinger L, Izazola JA, Loures L, DeLay P, Ghys PD. What Is Required to End the AIDS Epidemic as a Public Health Threat by 2030? The Cost and Impact of the Fast-Track Approach. PLoS One. 2016;11(5):e0154893 Epub 2016/05/10. doi: 10.1371/journal.pone.0154893 . 27159260 10.1371/journal.pone.0154893 PMC4861332

[10] Avenir Health. Goals manual: a model for estimating the effects of interventions and resource allocation on HIV infections and deaths. Glastonbury, CT: 2011 August. Report No.

[11] Newman L, Rowley J, VanderHoorn S, Wijesooriya NS, Unemo M, Stevens G, et al Global estimates of the prevalence and incidence of four curable sexually transmitted infections in 2012. PLoS One. 2015;10(12):e0143304 Epub Dec 8. doi: 10.1371/journal.pone.0143304 26646541 10.1371/journal.pone.0143304 PMC4672879

[12] Ham DC, Lin C, Newman L, Wijesooriya NS, Kamb M. Improving global estimates of syphilis in pregnancy by diagnostic test type: A systematic review and meta-analysis. Int J Gynaecol Obstet. 2015;130 Suppl 1:S10–4. Epub 2015/05/13. doi: 10.1016/j.ijgo.2015.04.012 . 25963909 10.1016/j.ijgo.2015.04.012 PMC4591031

[13] Gerbase AC, Rowley JT, Heymann DH, Berkley SF, Piot P. Global prevalence and incidence estimates of selected curable STDs. Sex Transm Infect. 1998;74 Suppl 1:S12–6. Epub 1999/02/19. . 10023347

[14] World Health Organization. Global incidence and prevalence of selected curable sexually transmitted infections—2008 Geneva: 2012.

[15] El Kettani A, Mahiané G, Abu-Raddad L, Smolak A, Rowley J, Nagelkerke N, et al Trends in adult chlamydia and gonorrhea prevalence, incidence and urethral discharge case reporting in Morocco over 1995 to 2015 –estimates using the Spectrum-STI model. Sex Transm Dis. In press. 10.1097/OLQ.0000000000000647 PMC5559184 28806354

[16] Ministère de la santé du Maroc. Etude sur les comportements, attitudes et pratiques des jeunes en matière de VIH-sida. Rabat: 2007.

[17] Ministère de la santé du Maroc. Etude sur les comportements, attitudes et pratiques des jeunes en matière de VIH-sida. Rabat: 2013.

[18] Alami K, Ait Mbarek N, Akrim M, Bellaji B, Hansali A, Khattabi H, et al [Urethral discharge in Morocco: prevalence of microorganisms and susceptibility of gonococcos]. East Mediterr Health J. 2002;8(6):794–804. Epub 2004/12/01. . 15568457

[19] Hancali A, Ndowa F, Bellaji B, Bennani A, Kettani A, Charof R, et al Antimicrobial resistance monitoring in Neisseria gonorrhoeae and strategic use of funds from the Global Fund to set up a systematic Moroccan gonococcal antimicrobial surveillance programme. Sex Transm Infect. 2013;89 Suppl 4:iv24–7. Epub 2013/09/17. doi: 10.1136/sextrans-2013-051166 . 24037141 10.1136/sextrans-2013-051166

[20] Mumtaz G, Hilmi N, Zidouh A, El Rhilani H, Alami K, Bennani A, et al. National and Souss Massa Draa HIV Modes of Transmission analysis in Morocco 2008. Rabat: Kingdom of Morocco Ministry of Health Department of Epidemiology and Disease Control National STI/AIDS Programme, Weill Cornell Medical College in Qatar, ONUSIDA, 2010 August. Report No.

[21] Kouyoumjian SP, Haddad P, El Rhilani H, Latifi A, El Kettani A, Alami K, et al. National and Souss Massa Draa HIV Modes of Transmission analysis in Morocco 2013. Rabat: Kingdom of Morocco Ministry of Health Department of Epidemiology and Disease Control National STI/AIDS Programme, Weill Cornell Medical College in Qatar, Qatar Foundation, ONUSIDA, 2014 January. Report No.

[22] Ministère de la Santé du Maroc. Estimations Nationales VIH /sida 2016: Rapport National 2016 sur la Mise en oeuvre de la declaration politique sur le VIH/SID Rabat: 2016.

[23] Royaume du Maroc Programme National de lutte contre les IST/SIDA Direction de l'épidémiologie et des luttes contre les maladies du Ministère de la Santé. Etude de prévalence IST chez les femmes consultantes en SMI/PF à la Wilaya de Rabat, Rapport final. Rabat: 2001.

[24] El Kettani A, Hançali A, Bennani A, Kharbach A, Alami K, Maaroufi A, editors. Prevalence of STIs in women seeking family planning and antenatal care in primary health care in Morocco. 17th IUSTI World Congress 2016; Marrakech.

[25] Korenromp EL, Mahiané G, El Kettani A, Hançali A, El Rhilani H, Alami K, et al. The Spectrum model estimating national syphilis and gonorrhea prevalence & trends: pilot application in Morocco. Marrakech: Morocco Service des MST-SIDA Direction de l'Epidemiologie et de Lutte Contre les Maladies (DELM) & Division des Maladies Transmissibles (DMT), World Health Organization Eastern Mediterranean office, UNAIDS Morocco country office, World Health Organization Headquarters, 2016 13–14 May. Report No.

[26] Korenromp EL, de Vlas SJ, Nagelkerke NJ, Habbema JD. Estimating the magnitude of STD cofactor effects on HIV transmission: how well can it be done? Sex Transm Dis. 2001;28(11):613–21. Epub 2001/10/26. . 11677381 10.1097/00007435-200111000-00001

[27] Wijesooriya NS, Rochat RW, Kamb ML, Turlapati P, Temmerman M, Broutet N, et al Global burden of maternal and congenital syphilis in 2008 and 2012: a health systems modelling study. Lancet Glob Health. 2016;4(8):e525–33. doi: 10.1016/S2214-109X(16)30135-8 27443780 10.1016/S2214-109X(16)30135-8 PMC6759483

[28] Ministère de la Santé [Maroc], ORC Macro, Ligue des États Arabes. Enquête sur la Population et la Santé Familiale (EPSF) 2003–2004. Calverton, Maryland, USA Ministère de la Santé et ORC Macro, 2005.

[29] Royaume du Maroc Programme National de lutte contre les IST/SIDA Direction de l'épidémiologie et des luttes contres les maladies du ministère de la santé. Etude de prévalence des IST chez les femmes qui consultent pour pertes vaginales et/ou douleurs du bas ventre. Rabat: 2008 June. Report No.

[30] Johnston LG, McLaughlin KR, El Rhilani H, Latifi A, Toufik A, Bennani A, et al Estimating the Size of Hidden Populations Using Respondent-driven Sampling Data: Case Examples from Morocco. Epidemiology. 2015;26(6):846–52. Epub 2015/08/11. . 26258908 10.1097/EDE.0000000000000362 PMC4586393

[31] South Africa Ministry of Health. National HIV and Syphilis Sero-Prevalence Survey of women attending Public Antenatal Clinics in South Africa 2000. Johannesburg: 2000.

[32] Ministère de la Santé du Maroc. Surveillance sentinelle du VIH et de la syphilis. Rabat: 2005–2012.

[33] Centers for Disease Control USA, ICAP project at Columbia University, Zimbabwe National AIDS Council (NAC), Zimbabwe National Statistics Agency (ZIMSTAT), Zimbabwe Biomedical Research and Training Institute (BRTI). Zimbabwe population-based HIV impact assessment ZIMPHIA 2015–2016. Summary sheet: preliminary findings. 2016 Contract No.: December.

[34] World Health Organization. Report on global sexually transmitted infection surveillance 2013. Geneva: 2014 June. Report No.

[35] World Health Organization. Baseline report on global sexually transmitted infection surveillance 2012. Geneva: 2013.

[36] World Health Organization. A tool for strengthening STI surveillance at the country level. Geneva: 2015 14 April. Report No.

