# Supplementary material for: The prevalence and incidence of active syphilis in women in Morocco, 1995-2016: Model-based estimation and implications for STI surveillance
Source: PLoS One. 2017 Aug 24;12(8):e0181498. doi: 10.1371/journal.pone.0181498 (PMC5570350; doi:10.1371/journal.pone.0181498)
Supplement: S1 Text — (DOCX) [file pone.0181498.s001.docx]

# **S1 text. Derivation of the incidence hazard and rate, from prevalence**

Let us consider a Susceptible-Infected-Susceptible model. This allows describing the dynamic of a curable infection for which cured individuals become susceptible e.g. syphilis. Let S represent the susceptible population and I the infected one. Let us denote the incidence hazard rate (among uninfected people) and the recovery rate by $i$ and$r$, respectively, where$r=1/D$, D being the duration of infection. The dynamics of the infection in our population can be described by the system:

$$\begin{matrix} dS/dt & = & -iS-\mu S+rI \\ dI/dt & = & iS-\left( \mu+r \right)I \end{matrix} Eq.S1$$

where $\mu$ is the (background, non-STI-related) mortality rate.

Equations similar to Eq.S1 are often used to describe the spread of infectious diseases in populations [[1](#_ENREF_1)].

Now, because prevalence is given by$p=I/(I+S)$, one can use Eq.S1 to show that the prevalence satisfies the equation ${dp}/{dt}=i-\left( i+r \right)p$. This shows that the prevalence does not depend on the (background) mortality. Solving the latter ordinary differential equation gives Eq1 in the main text, which describes the relation between the incidence hazard rate, the prevalence and the recovery rate:

$p\left( t \right)=\frac{i}{i+r}+\left( p\left( t_{0} \right)-\frac{i}{i+r} \right)exp\left( -\left( i+r \right)\left( t-t_{0} \right) \right)$, for all$t\geq t_{0}$. When the prevalence (at times $t_{0}$ and$t$), and the recovery rate are known, solving Eq1 for $i$ gives the incidence hazard rate in the interval ($t_{0},t$). That equation is non-linear but can be solved numerically, using a Newton type algorithm. If $t-t_{0}$ is not very large, a good starting point is$i_{0}=\left( 2\left( p_{1}-p_{0} \right)+r\left( p_{1}+p_{0} \right) \right)/\left( 2-p_{1}-p_{0} \right)$, where $p_{0}=p\left( t_{0} \right)$ and$p_{1}=p\left( t \right)$.

The assumed durations of STI episodes (from the WHO 2012 regional and global estimation [[2](#_ENREF_2)], as averages between treated and untreated episodes, in years) are given in Table S1.

**References for S1 text:**

1. White R, Vinnicky E. An Introduction to Infectious Diseases Modelling. 1st ed. Oxford: Oxford University Press, USA; 2010.

2. Newman L, Rowley J, VanderHoorn S, Wijesooriya NS, Unemo M, Stevens G, et al. Global estimates of the prevalence and incidence of four curable sexually transmitted infections in 2012. PLoS One. 2015;10(12):e0143304. Epub Dec 8. doi: 10.1371/journal.pone.0143304.
