# Supplementary material for: The prevalence and incidence of active syphilis in women in Morocco, 1995-2016: Model-based estimation and implications for STI surveillance
Source: PLoS One. 2017 Aug 24;12(8):e0181498. doi: 10.1371/journal.pone.0181498 (PMC5570350; doi:10.1371/journal.pone.0181498)
Supplement: S1 Table — (DOCX) [file pone.0181498.s002.docx]

# **S1 Table. Duration of adult syphilis infection, in years**

|  |  | **Treatment access region** | | |
| --- | --- | --- | --- | --- |
|  | **Infection stage** | **A. Australasia, high-income North America & Asia Pacific, Western Europe** | **B.  Americas excluding North America, Oceania, Central Asia, Central Europe, Eastern Mediterranean (incl. Morocco)** | **C. Sub-Saharan Africa, South & South-East Asia** |
| Duration if treated, by stage at which treated | Primary | 1 month | | |
|  | Secondary | 3 months | | |
|  | Latent | 3 years | | |
| Duration of infection, when untreated | Tertiary | 15 years | | |
| % of episodes symptomatic | Primary | 40% | | |
|  | Secondary | 60% | | |
| Weighted average duration of (active) infection (2012) | | 1.28 years | 2.42 years | 4.13 years |
| % of symptomatic episodes treated (2012) | | 85% | 60% | 35% |

Legend to S1 Table: The Spectrum-STI model uses these durations to estimate an annual incidence rate from annual prevalence. The weighted average duration is calculated by weighing the durations for treated and untreated fractions, by their respective proportions of episodes, in turn calculated from the proportions of infections that are symptomatic and treated. All assumptions are taken as equal for adult women and adult men.
