# Supplementary material for: The prevalence and incidence of active syphilis in women in Morocco, 1995-2016: Model-based estimation and implications for STI surveillance
Source: PLoS One. 2017 Aug 24;12(8):e0181498. doi: 10.1371/journal.pone.0181498 (PMC5570350; doi:10.1371/journal.pone.0181498)
Supplement: S2 Table — (DOCX) [file pone.0181498.s003.docx]

# **S2 Table. Syphilis prevalence (RPR, without TPHA confirmation) in sentinel surveillance in non-ANC groups, Morocco**

Table S2 shows syphilis prevalence measured in sentinel surveillance, for all population groups sampled except for pregnant women in ANC services, for whom data are shown in Table 1. These data (except for ANC women) are not used for the Spectrum estimation, but serve as context to triangulate with the Spectrum-estimated trends.

For example, blood donor data are shown here (but not used for comparison in the main paper), because they are less likely to be representative of the general population, given possible self-selection (even if not paid) and because Morocco’s transfusion services exclude adults with self-reported risk behaviours [[1](#_ENREF_1)].

**Reference for S2 table:**

1. Maroc Ministère de la Santé Publique. Décret n° 2-94-20 (22 joumada II 1416) 16 novembre 1995 pris pour l'application de la loi n° 03-94 relative au don, au prélèvement et à l'utilisation du sang humain 1995.

**S2 Table.**

| **Year** | **Sentinel group** | **N tested** | **N RPR+** | **% RPR+** | **N TPHA+** | **% TPHA+** |
| --- | --- | --- | --- | --- | --- | --- |
| 2000 | STI patients | 4,765 | 282 | **5.92%** |  |  |
| 2004 | STI patients | 7,047 | 269 | **3.82%** |  |  |
| 2005 | STI patients | 5,343 | 263 | **4.92%** |  |  |
| 2007 | STI patients | 8,034 | 240 | **2.99%** | 307 | **3.82%** |
| 2010 | STI patients | 9,007 | 389 | **4.32%** | 381 | **4.23%** |
| 2012 | STI patients | 7,219 | 160 | **2.22%** | 189 | **2.62%** |
| 2000 | TB patients | 849 | 31 | **3.65%** |  |  |
| 2002 | TB patients | 1,408 | 58 | **4.12%** |  |  |
| 2004 | TB patients | 1,164 | 27 | **2.32%** |  |  |
| 2005 | TB patients | 1,026 | 41 | **4.00%** |  |  |
| 2007 | TB patients | 537 | 18 | **3.35%** | 12 | **2.23%** |
| 2010 | TB patients | 1,558 | 33 | **2.12%** | 36 | **2.31%** |
| 2012 | TB patients | 1,116 | 17 | **1.52%** | 17 | **1.52%** |
| 2000 | Taxi drivers | 432 | 2 | **0.46%** |  |  |
| 2007 | Taxi drivers | 483 | 3 | **0.62%** | 4 | **0.83%** |
| 2002 | Male prisoners | 1,782 | 115 | **6.48%** |  |  |
| 2004 | Male prisoners | 2,289 | 110 | **4.81%** |  |  |
| 2005 | Male prisoners | 1,638 | 66 | **4.03%** |  |  |
| 2007 | Male prisoners | 2,095 | 66 | **3.15%** | 120 | **5.73%** |
| 2010 | Male prisoners | 3,176 | 130 | **4.09%** | 148 | **4.66%** |
| 2012 | Male prisoners | 2,262 | 69 | **3.05%** | 83 | **3.67%** |
| 2002 | Female prisoners (excluding FSW) | 138 | 3 | **2.17%** |  |  |
| 2004 | Female prisoners (excluding FSW) | 167 | 13 | **7.78%** |  |  |
| 2005 | Female prisoners (excluding FSW) | 61 | 10 | **16.39%** |  |  |
| 2007 | Female prisoners (excluding FSW) | 95 | 5 | **5.26%** | 9 | **9.47%** |
| 2010 | Female prisoners (excluding FSW) | 274 | 27 | **9.85%** | 33 | **12.04%** |
| 2012 | Female prisoners (excluding FSW) | 334 | 12 | **3.59%** | 14 | **4.19%** |
| 2004 | Female sex workers, incarcerated | 332 | 32 | **9.64%** |  |  |
| 2005 | Female sex workers, incarcerated | 102 | 12 | **11.76%** |  |  |
| 2007 | Female sex workers, incarcerated | 228 | 22 | **9.65%** | 12 | **5.26%** |
| 2010 | Female sex workers, incarcerated | 195 | 20 | **10.26%** | 20 | **10.26%** |
| 2000 | New recruits | 398 | 2 | **0.50%** |  |  |
| 2004 | New recruits | 705 | 2 | **0.28%** |  |  |
| 2000 | Adults requesting a health insurance card | 202 | 2 | **0.99%** |  |  |
| 2004 | Adults requesting a health insurance card | 56 | - | **0.00%** |  |  |
| 2010 | Adults requesting a health insurance card | 269 | 7 | **2.60%** | 7 | **2.60%** |

**S2 Table** (continued)

| **Year** | **Sentinel group** | **N tested** | **N RPR+** | **% RPR+** | **N TPHA+** | **% TPHA+** |
| --- | --- | --- | --- | --- | --- | --- |
| 2004 | Hotel employees | 710 | 3 | **0.42%** |  |  |
| 2005 | Hotel employees | 847 | 8 | **0.94%** |  |  |
| 2007 | Hotel employees | 396 | 4 | **1.01%** | 4 | **1.01%** |
| 2004 | Truck drivers | 189 | 3 | **1.60%** |  |  |
| 2005 | Truck drivers | 199 | 19 | **9.55%** |  |  |
| 2010 | Truck drivers | 202 | 5 | **2.48%** | 5 | **2.48%** |
| 2012 | Truck drivers | 209 |  | **0.00%** | 0 | **0.00%** |
| 2004 | Fishermen | 544 | 11 | **1.97%** |  |  |
| 2005 | Fishermen | 356 | 12 | **3.37%** |  |  |
| 2007 | Fishermen | 147 | 2 | **1.36%** | 1 | **0.68%** |
| 2010 | Fishermen | 730 | 24 | **3.29%** | 24 | **3.29%** |
| 2012 | Fishermen | 617 | 16 | **2.59%** | 16 | **2.59%** |
| 2004 | Female seasonal workers | 311 | 9 | **2.89%** |  |  |
| 2005 | Female seasonal workers | 138 | 3 | **2.17%** |  |  |
| 2007 | Female seasonal workers | 398 | 17 | **4.27%** | 16 | **4.02%** |
| 2010 | Female seasonal workers | 228 | 4 | **1.75%** | 4 | **1.75%** |
| 2012 | Female seasonal workers | 128 | - | **0.00%** | 0 | **0.00%** |
| 2004 | Female factory workers | 19 | - | **0.00%** |  |  |
| 2005 | Female factory workers | 133 | 6 | **4.51%** |  |  |
| 2010 | Female factory workers | 43 | 3 | **6.98%** | 3 | **6.98%** |
| 2012 | Female factory workers | 106 | 4 | **3.77%** | 4 | **3.77%** |
| 2010 | Men having sex with men | 95 | 16 | **16.84%** | 16 | **16.84%** |
| 2012 | Men having sex with men | 163 | 1 | **0.61%** | 2 | **1.23%** |
| 2010 | Intravenous Drug Users | 65 | 4 | **6.15%** | 4 | **6.15%** |
| 2012 | Intravenous Drug Users | 115 | 5 | **4.35%** | 5 | **4.35%** |
